# Supplementary material for: Percutaneous Endoscopic Gastrostomy and Nutritional Interventions by the Pediatric Nutritional Support Team Improve the Nutritional Status of Neurologically Impaired Children
Source: J Clin Med. 2020 Oct 14;9(10):3295. doi: 10.3390/jcm9103295 (PMC7602143; doi:10.3390/jcm9103295)
Supplement: Supplementary file 1 [file jcm-09-03295-s001.pdf]

Supplementary Table S1. Laboratory findings during the study period.

	Median (IQR)			*p*-value	
	Initial Visit	6-month Visit	Latest Visit	*	**
Hb	11.65 (10.3–12.9)	12.3 (10.9–13.4)	12.35 (11.0–14.0)	0.209	0.530
Lymphocyte	1932 (1351.25–2817.75)	1870 (1162–3141.5)	2596 (1889.25–3301.50)	0.463	0.594
Protein	6.80 (5.88–7.43)	7.0 (6.65–7.65)	6.85 (6.6–7.25)	0.224	0.234
Albumin	3.70 (3.40–3.83)	3.90 (3.45–4.20)	3.65 (3.38–4.05)	0.223	0.161
BUN	7.7 (5.2–12.05)	11.15 (6.53–12.83)	9.2 (5.2–11.69	0.495	0.506
Calcium	9.30 (8.88–9.63)	9.10 (8.65–9.65)	8.90 (8.68–9.239	0.161	0.003
Ca/P ratio	2.09:1 (1.73:1–2.38:1)	2.04:1 (1.87:1–2.5:1)	2.30:1 (2.03:1–2.88:1)	0.334	0.100
25-OH-VitD3	29.60 (17.60–38.45)	18.10 (12.70–31.60)	28.40 (16.40–39.40)	0.263	0.123

p-values were determined using the Wilcoxon signed-rank test. * *p*-value for differences in the laboratory data between the initial visit and the 6-month visit. ** *p*-value for differences in the laboratory data between the initial visit and the latest visit. BUN, blood urea nitrogen; Hb, hemoglobin; IQR, interquartile range.

Supplementary Table S2. Comparisons of the anthropometric and nutritional parameters between the previous oral feeding group and NG tube feeding group during the study period.

Measurement		Type	Median (IQR)			*p*-values		
			Baseline Visit (Oral Group = 8, NG Tube Group = 10)	6-month Visit (Oral Group = 7, NG Tube Group = 9)	Latest Visit (Oral Group = 6, NG Tube Group = 8)	*	**	***
Height	Percentile [a)]	Oral	68.76 (47.07–79.8)	54.17 (45.91–73.62)	60.81 (33.6–85.57)	*0.50*	*0.50*	*0.35*
		NG tube	57.9 (17.51–82.11)	55.96 (17.65–79.33)	53.48 (28.66–84.86)	*0.07*	*0.50*	*0.33*
		p-value	*0.53*	*0.71*	*0.70*			
	z-score [b)]	Oral	−4.39 (−5.59–0.24)	-4.22 (-6.07 to 0.51)	-2.93 (-5.51 to -0.78)	*0.74*	*0.14*	*0.17*
		NG tube	−2.46 (−3.81–(−0.73))	−2.75 (−4.57 to −0.62)	−2.62 (−3.83 to −1.08)	*0.17*	*0.09*	*0.40*
		p-value	*0.66*	*0.63*	*0.80*			
Weight	Percentile [a)]	Oral	21.38 (5.62–40.48)	14.89 (4.07–64.29)	29.73 (11.23–51.35)	*0.09*	*0.50*	*0.46*
		NG tube	51.54 (13.81–80.66)	61.59 (20.34–82.64)	78.57 (40.66–86.67)	*0.51*	*0.75*	*0.48*
		p-value	*0.06*	*0.12*	*0.05*			
	z-score [b)]	Oral	−5.71 (−7.52–(−2.88))	−4.99 (−7.59 to −0.1)	−4.7 (−6.09–(−1.51))	*0.02*	*0.22*	*0.92*
		NG tube	−1.62 (−6.44–0.36)	−1.75 (−5.58–1.01)	−1.39 (−4.33–0.94)	*0.26*	*0.31*	*0.89*
		p-value	*0.13*	*0.22*	*0.12*			
BMI	Percentile [a)]	Oral	4.64 (4.41–5.05)	4.79 (3.81–25.64)	4.8 (4.13–40.87)	*0.46*	*0.07*	*0.50*
		NG tube	30.93 (11.55–47.17)	50.04 (11.38–59.4)	55.48 (16.07–66.43)	*0.21*	*0.07*	*0.24*

	***p*-value**	*0.02*	*0.03*	*0.06*				
**z-score b)**	**Oral**	−4.55 (−5.97–(−3.85))	−3.49 (−4.64–(−0.83))	−3.02 (−4.67–(−0.9))	*0.02*	*0.47*	*0.05*
	**NG tube**	−1.13 (−3.2–0.28)	−0.9 (−2.73–2.11)	−0.6 (−3.21–3.29)	*0.09*	*0.87*	*1.00*
	***p*-value**	*0.02*	*0.12*	*0.25*			
**PIBW**	**Oral**	65.19 (49.99–80.2)	77.42 (66.78–87.64)	73.82 (66.03–94.01)	*0.03*	*0.50*	*0.35*
	**NG tube**	91.6 (78.01–97.94)	102.16 (80.45–109.82)	106.91 (79.33–131.19)	*0.05*	*0.03*	*0.12*
	***p*-value**	*0.03*	*0.10*	*0.07*			
**CIR**	**Oral**	43.55 (38.75–49.56)	81.67 (80–100)	89.59 (80–100)	*0.01*	*1.00*	*0.05*
	**NG tube**	62.5 (34.78–92.32)	80 (58.13–95.84)	84.53 (68.95–91.75)	*0.48*	*0.92*	*0.21*
	***p*-value**	*0.72*	*0.33*	*0.24*			
**PIR**	**Oral**	50.12 (38.34–56.28)	88.34 (72.5–110.67)	90 (85.63–95.91)	*0.02*	*0.69*	*0.03*
	**NG tube**	55.68 (29.7–88.79)	79.34 (61.12–96.2)	88.34 (77.84–97.5)	*0.11*	*0.50*	*0.03*
	***p*-value**	*0.53*	*0.25*	*0.56*			

p-values were determined using the Wilcoxon signed−rank test. * *p*-value for differences between the initial visit and the 6−month visit. ** *p*-value for differences between the 6−month visit and the latest visit. *** *p*-value for differences between the initial visit and the latest visitBMI, body mass index; CIR, ratio of calorie intake to required amount; IQR, interquartile range; NG, nasogastric; PIBW, percent of ideal body weight; PIR, ratio of protein intake to required amount. a) Percentile was measured based on the growth curves for cerebral palsy according to sex and Gross Motor Function Classification System level. b) z−score was calculated based on the 2017 Korea National Growth Chart data

Supplementary Table S3. Comparisons of the anthropometric and nutritional parameters between the hypertonic and hypotonic groups during the study period.

Measurement		Type	Median (IQR)			*p*-Values		
			**Baseline Visit (Hypertonic Type = 14, Hypotonic Type = 4)**	**6−month Visit (Hypertonic Group = 12, Hypotonic Group = 4)**	**Latest visit (Hypertonic Group = 11, Hypotonic Group = 3)**	*	**	***
**Height**	**Percentile a)**	**Hypertonic**	58.66 (36.99–82.11)	53.05 (25.89–80.6)	60.06 (35.27–82.55)	*0.25*	*0.31*	*0.37*
		**Hypotonic**	76.98 (44.53–79.8)	69.08 (40.05–72.49)	39.29 (28.58–94.63)	*0.07*	*0.59*	*0.59*
		***p*-value**	*0.67*	*0.72*	*0.94*			
	**z−score b)**	**Hypertonic**	−2.88 (−5.64–(−0.78))	−3.25 (−5.94–(−0.95))	−2.41 (−4.98–(−1.46))	*0.10*	*0.05*	*0.18*
		**Hypotonic**	−2.54 (−5.25–0.86)	−2.35 (−4.68–0.74)	−3.46 (−4.36–1.28)	*0.72*	*0.59*	*0.59*
		***p*-value**	*0.60*	*0.47*	*0.82*			
**Weight**	**Percentile a)**	**Hypertonic**	38.92 (11.7–75.92)	41.39 (13.15–78.91)	54.8 (14–82.76)	*0.35*	*0.78*	*0.48*
		**Hypotonic**	21.95 (4.03–50.15)	32.83 (4.05–71.65)	47.5 (4.05–87.59)	*0.07*	*0.59*	*0.59*
		***p*-value**	*0.20*	*0.28*	*0.82*			

	**z−score** b)	**Hypertonic**	−3.85 (−6.67–(−1.17))	−3.61 (−6.21–0.6)	−3.42 (−5.55– (−0.65))	*0.07*	*0.11*	*0.86*
		**Hypotonic**	−3.42 (−9.06–0.24)	−2.69 (−8.26– 0.78)	−1.2 (−5.36–1.17)	*0.07*	*0.59*	*0.59*
		***p*-value**	*0.92*	*0.81*	*0.48*			
**BMI**	**Percentile** a)	**Hypertonic**	22.6 (4.73–39.6)	26.8 (4.66–56.42)	41.22 (4.61–57.17)	*0.15*	*0.03*	*0.17*
		**Hypotonic**	4.52 (3.53–18.23)	3.96 (3.37–31.54)	4.94 (4.13–90.76)	*1.00*	*0.18*	*0.65*
		***p*-value**	*0.12*	*0.10*	*0.87*			
	**z−score** b)	**Hypertonic**	−3.29 (−4.51–(−0.43))	−1.18 (−3.93–1.33)	−1.63 (−4.52–0.44)	*0.03*	*0.59*	*0.42*
		**Hypotonic**	−2.93 (−9.31–(−0.55))	−2.41 (−7.75–0.46)	0.71 (−4.46–4.13)	*0.07*	*0.18*	*0.11*
		***p*-value**	*0.52*	*0.63*	*0.39*			
**PIBW**		**Hypertonic**	86.51 (65.49–93.79)	90.44 (73.14–103.77)	91.29 (75.34–108.67)	*0.02*	*0.02*	*0.05*
		**Hypotonic**	63.28 (45.7–92.95)	81.57 (57.67–103.91)	69.13 (68.97–138.68)	*0.07*	*0.59*	*0.59*
		***p*-value**	*0.29*	*0.47*	*0.70*			
**CIR**		**Hypertonic**	43.13 (37.22–84.94)	83.34 (67.5–100)	85.72 (77.78–100)	*0.07*	*0.92*	*0.03*
		**Hypotonic**	47.03 (33.96–76.34)	80 (76.25–95)	85.72 (80–91.67)	*0.07*	*1.00*	*0.29*
		***p*-value**	*1.00*	*0.86*	*0.94*			
**PIR**		**Hypertonic**	50.3 (35.84–84.32)	85.72 (70.78–111.12)	90 (80–100)	*0.01*	*0.59*	*0.01*
		**Hypotonic**	46.12 (31.6–74.42)	71 (68.5–90)	90 (87.5–90)	*0.14*	*0.29*	*0.11*
		***p*-value**	*0.60*	*0.37*	*0.94*			

p-values were determined using the Wilcoxon signed−rank test. * *p*-value for differences between the initial visit and the 6−month visit. ** *p*-value for differences between the 6−month visit and the latest visit. *** *p*-value for differences between the initial visit and the latest visit. BMI, body mass index; CIR, ratio of calorie intake to required amount; IQR, interquartile range; PIBW, percent of ideal body weight; PIR, ratio of protein intake to required amount. a) Percentile was measured based on the growth curves for cerebral palsy according to sex and Gross Motor Function Classification System level. b) *z*−score was calculated based on 2017 Korea National Growth Chart data.
